# The confluence of radiotherapy and immunotherapy

**DOI:** 10.3389/fonc.2012.00143

**Published:** 2012-10-16

**Authors:** Byron Burnette, Yang-Xin Fu, Ralph R. Weichselbaum

**Affiliations:** ^1^Department of Pathology, The Ludwig Center for Metastasis Research, The University of ChicagoChicago, IL, USA; ^2^Department of Radiation and Cellular Oncology, The Ludwig Center for Metastasis Research, The University of ChicagoChicago, IL, USA

**Keywords:** radiation, immunotherapy, radiotherapy, T cell, danger signal

## Abstract

Radiotherapy (RT) has been considered a local modality and outcomes have emphasized local and regional control of tumors. Recent data suggests that RT may activate the immune system and the combination of radiation therapy and immune therapies may have the potential to improve both local and distant control of tumor deposits. Below we review principals underlying the concepts of combining both modalities.

## INTRODUCTION

The utility of radiotherapy (RT) as an anti-tumor agent is usually based on the fact that radiation can induce irreparable DNA damage, and eventually cell death through a variety of mechanisms including; mitotic catastrophe, apoptosis, senescence, and autophagy (Rupnow and Knox, 1999; Eriksson and Stigbrand, 2010). Improvements in the clinical practice of RT were historically aimed at technically achieving maximal tumor cell killing while balancing damage to normal tissues. However, over the past decade RT has been the subject of a steady conceptual and experimental reinvention that has broadened both our understanding of the mechanisms by which RT mediates tumor eradication and possibilities for synergistic combinations with emerging anti-cancer therapies. Of particular relevance to this review is the finding that in a variety of preclinical animal models adaptive immunity plays a defining role in the efficacy of RT (Lugade et al., 2005; Lee et al., 2009). The mechanisms underlying the capacity of RT to engage the immune system are the subject of intense scientific inquiry. Published data demonstrate that RT can induce or augment all phases of the T cell response from T cell priming, trafficking, and effector responses within the tumor, which endorses a natural alignment of radiation and immunotherapy. The data from preclinical models may overemphasize the role of adaptive immunity in RT as a single modality, which may explain the paucity of supporting clinical data. Only relatively recently has there been a meaningful effort to assess immunological correlates in the course of traditional RT. Regardless of the overall contribution of adaptive immunity to RT, at the very least the immune system is poised to be a powerful ally with a demonstrated capacity to augment the anti-tumor effects of RT. Therefore, several aspects of clinical RT warrant reconsideration with respect to the role of endogenous anti-tumor immunity especially in light of combinatorial treatment strategies that incorporate immunotherapy. In this review, we will discuss these and other aspects of RT that could affect the proposed synergistic relationship between RT and immunotherapy and also highlight some novel strategies that aim to further exploit the immunogenicity of RT.

## IMMUNE RECOGNITION OF TUMORS

The principals of tumor immunology were originally established by pioneering work of Burnet and Thomas when they proposed that nascent tumors can be recognized and eliminated by the host immune system in a process they termed “cancer immunosurveillance” (reviewed in Dunn et al., 2006). By inference, immunosurveillance governs the capacity of the immune system to “recognize” the tumor. From simplified viewpoint, this interaction can be divided into two processes whereby the immune system is first “alerted” to the presence of cells undergoing neoplastic transformation through stress or danger signals, and second, is equipped to directly interact with neoplastic cells to mediate destruction. Although considerable debate still exists regarding whether immunosurveillance exists in human and mouse tumors, the underlying principles that define the capacity of the immune system to specifically recognize tumors remain unchanged. Therefore, whether or not the emergence of clinically detectable tumors is reduced by immune-mediated mechanisms does not preclude subsequent immune recognition that could occur during the clinical treatment of tumors. A logical extension of the principles of cancer immunosurveillance, therefore, lies in the hypothesis that successful treatment of established tumors, as potential products of failed or blunted surveillance, could be achieved by rekindling immune recognition. This hypothesis is the foundation of the field of tumor immunology and its applied counterpart cancer immunotherapy. Cancer immunotherapy represents the use of agents proposed to amplify the host immune response to established tumors (Pardoll and Drake, 2012). Radiation therapy and immunotherapy may be natural partners given that radiation possesses immunomodulatory effects at multiple points in the processes of T cell priming and effector function. We will review literature regarding the immunomodulatory properties of radiation and discuss available data dealing with the effect of dose and fractionation schedules on various aspects of the anti-tumor immune response.

## EFFECTS ON TUMOR ANTIGENICITY

The first major requirement for tumor-specific adaptive immunity is the availability and immunogenicity of tumor antigens. A plethora of tumor antigens have been defined across a wide array of tumor types and they fall into three broad categories: (1) viral proteins, (2) mutated versions of self-proteins that include point mutations and oncogenic fusion proteins generated by recombinatorial events, or (3) non-mutated self-proteins enriched in tumor cells but with shared expression on non-tumor tissue (for review, see Jäger et al., 2001). Melanoma differentiation antigens and cancer testis (CT) antigens are the best characterized tumor-associated antigens (Engelhard et al., 2002; Scanlan et al., 2002). The etiology of tumor antigens has important implications on immunogenicity. Non-mutated tumor-associated antigens are self-antigens that are subject to immunological tolerance mechanism that drastically diminish the peripheral repertoire of high-affinity T cells capable of recognizing these antigens. However, tumor-associated antigens offer a convenient clinical target both for therapeutic vaccination and immunological assessment due to a high frequency of expression across many tumor types. Mutated tumor antigens represent the most unique antigens that, based on their extrathymic expression, would be excluded from central tolerance. Therefore, T cells expressing high-affinity T cell receptors (TCRs) specific for these antigens are likely to be present in the peripheral pool. Identification and vaccination against such antigens, however, requires sophisticated high-throughput screening methods to identify mutations and sift out the potential antigenic peptides with sufficient binding to major histocompatibility (MHC) antigens to mediate efficient presentation. Notable exceptions that can be readily identified are antigens generated by mutated oncogenic proteins that have high association with some cancers (Boon, 1996). Non-mutated tumor-associated antigens, on the other hand, are readily identified by established screening methods and are widely expressed across tumor types. Such antigens are often accompanied by some degree of T cell tolerance that dampens endogenous immunity (Engelhard et al., 2002). Nevertheless, clinically viable vaccination strategies have been developed that can induce durable T cell responses against tumor-associated antigens, and even low-avidity T cells that escape negative selection can mediate anti-tumor effects if properly activated (Uchi et al., 2006).

With regard to tumor-antigen expression, local high dose ablative (15–20 Gy) radiation has been shown to directly upregulate the expression of some tumor antigens including tumor antigens associated with viral transformation (Santin et al., 1998), and CT antigens (Sharma et al., 2011). A mechanistic basis for these changes was reported by Reits et al. (2006) who demonstrated that tumor cell irradiation leads to increased protein translation as a consequence of mTOR activation. Furthermore, radiation increased the degradation of cellular proteins as a result of direct free radical-mediated damage. The resulting increase in the intracellular pool of available peptides augments MHC loading and productive antigen presentation. Interestingly, dose-dependent effects of radiation were observed in terms of both the magnitude and duration of intracellular peptide availability. Single doses of higher than 4 Gy were required to dramatically enhance MHC class I surface expression, and a single dose of 25 Gy induced the most robust expression, which correlated with measurements of intracellular peptide levels. Together these mechanisms could overcome the poor antigenicity of some tumors in instances where availability of tumor antigens is a limiting factor to the induction of tumor antigen-specific T cells. Although, the mechanisms uncovered by Reits et al. (2006) provide an interesting mechanism by which local RT could enhance local T cell-mediated recognition, whether or not this mechanism plays any role in augmenting endogenous immunity remains unknown. It would be interesting to know whether the intermediate effect of local radiation alone on tumor growth in their model could be abrogated by systemic CD8 T cell depletion. Nevertheless, these studies suggest that larger doses of radiation are more potent at increasing tumor antigenicity, however, the effect of smaller daily fractionated doses was not investigated. It is possible that daily doses of less than 2 Gy, such as those used in traditional RT, might eventually result in a cumulative effect that could eventually approach the large single doses used by the authors given the kinetics of increased protein degradation. Notably, if the effect of radiation on MHC class I surface expression is indeed mediated through alleviation of the normally limiting pool of available intracellular peptides, then these effects would presumably be unrelated to intrinsic tumor cell radiosensitivity and therefore uniform across most tumor cells unless the efficiency of targeted protein degradation varies widely. In order to make these peptides available as substrates for T cell priming, however, transfer to professional antigen presenting cells (APCs) must occur in such a manner that stimulates efficient capture and intracellular processing within the APC to yield MHC:peptide complexes that are subsequently presented to T cells, a process termed antigen cross-presentation.

## RADIATION-MEDIATED “DANGER SIGNALS” AND CROSS-PRESENTATION OF TUMOR ANTIGENS

The innate immune system is equipped with many molecular sensors that facilitate the recognition of unique molecular patterns found in the myriad pathogens present in the environment. These sensors are localized to subcellular locations including the plasma membrane, endosomes, and the cytoplasm poised to detect invading pathogens. Charles Janeway proposed a cellular recognition system, consisting of receptors that could exclusively recognize unique features of pathogens (pathogen-associated molecular patterns, PAMPs), that formed the central basis for “self” vs “non-self” discrimination. This pathogen recognition system was thought to explain why antigens derived from pathogens elicit potent adaptive immune responses, and self-antigens are “ignored.” A highly provocative amendment to this hypothesis was proposed by Polly Matzinger, who hypothesized that tissue injury in the absence of pathogens could elicit innate immune recognition through stress signals that she collectively termed “Danger Signals” (Matzinger, 1994). It is now recognized that many of these same receptors do double duty and can recognize endogenous molecular signals emanating from stressed or dying cells in the absence of pathogens (Matzinger, 2002). Thus, the host actually senses “danger” in the form of cellular stress and tissue injury rather than sensing the presence of a pathogen specifically. The endogenous ligands are termed molecular “alarmins” and together with PAMPs they are collectively termed “danger signals” (Bianchi, 2006). Alarmins function as endogenous adjuvants that form an essential bridge between innate inflammatory responses and the initiation of tumor-specific adaptive immunity following treatment of tumors with local radiation. The exposure or release of danger signals also depends on the type of cell death that occurs and many open questions remain regarding the relative contributions of each to the immunogenicity of radiation-mediated tumor cell death.

High mobility group box 1 (HMGB1) is a prototypical “alarmin” that was shown to be a central mediator in the immunogenicity of dying tumor cells following irradiation (Apetoh et al., 2007). Normally a nuclear protein associated with chromatin, extracellular release of HMGB1 from dying tumor cells was demonstrated to engage Toll-like receptor 4 (TLR4) expressed by dendritic cells (DCs) to facilitate their activation, maturation, and capacity to efficiently prime tumor antigen-specific CD8^+^ T cells (T cell cross-priming). Since TLR4 binds ligands at the plasma membrane, HMGB1 must be released into the extracellular space in order to engage TLR4. Extracellular exposure could be mediated by direct necrotic cell death or secondary necrosis of lingering apoptotic bodies that are inefficiently cleared. Conceptually, apoptotic death of tumor cells is predicted to conceal HMGB1 from TLR4-mediated recognition (Bianchi and Manfredi, 2007). However radiation-mediated cell death of most solid tumors is thought to predominantly occur through induction of senescence, necrosis, or mitotic catastrophe. An exception is hematopoietic tumors that frequently undergo rapid induction of apoptosis following radiation exposure (Rupnow and Knox, 1999; Eriksson and Stigbrand, 2010). Radiation-mediated mitotic death shares features with both apoptosis and necrosis, however, the prevailing view places it more closely associated with necrosis. More recently, a specific receptor for necrotic cells was cloned and characterized. DNGR-1/CLEC9A, a c-type lectin, was shown to be essential for the induction of adaptive immunity to necrotic cells (Sancho et al., 2009). Importantly, interaction of DNGR-1 with necrotic cells did not affect the uptake of necrotic debris, but instead regulated the capacity of DCs to cross-present antigens contained therein. Furthermore, DNGR-1 expression was shown to specifically identify mouse and human DCs that express the transcription factor Batf3 and are specialized for cross-presentation of antigens to CD8^+^ T cells (Poulin et al., 2010, 2012; Schreibelt et al., 2012). The ligand for DNGR-1 was recently identified to be filamentous actin (F-actin) that is exposed upon the loss of membrane integrity characteristic of necrotic cell death (Ahrens et al., 2012). These concepts may be related to radiation induction of antigen processing and remain to be studied.

In addition to ligands that promote DC activation and subsequent maturation, several other danger signals contribute to immunological recognition of dying tumor cells. In particular, nucleotides released by apoptotic cells function as a chemotactic signal for phagocytic myeloid cells including DCs by stimulating the P2RY2 purinergic receptor (Elliott et al., 2009). Extracellular ATP can also function through P2RX7 purinergic receptors to initiate NLRP3 inflammasome activation and subsequent IL-1β production that were all shown to be required for the induction of tumor antigen-specific CD8^+^ T cells following challenge with dying tumor cells (Ghiringhelli et al., 2009). Finally, surface translocation of the ER resident protein calreticulin (together with ERP57) was shown to be an essential signal for efficient uptake of dying tumor cells by APCs and therefore a critical regulator of immunogenic cell death following tumor cell exposure to γ-irradiation (Obeid et al., 2006, 2007). Calreticulin exposure proceeds as a preapoptotic event that could be partially blocked by caspase inhibition, however, it is unclear whether translocation of calreticulin is a widely observed phenomenon across all modes of cell death induced by irradiation or if it is unique to cells destined to undergo apoptosis.

Taken together, the radiation-induced release of tumor antigens must be accompanied by coincident release and recognition of danger signals in order to efficiently generate tumor-specific CTLs. The ability of radiation to promote tumor antigen release has been demonstrated in several models, however, the type and magnitude of danger signal release is quite variable and may still provide suboptimal maturation signals to APCs. The coadministration of exogenous danger signals in the context of tumor irradiation has been shown to augment the immunogenicity of RT in both preclinical animal models and clinical trials. In particular, treatment of mice with a synthetic TLR9 agonist resulted in both enhanced local control and reduced distant metastasis when combined with single high dose RT (Zhang et al., 2012). Augmented tumor control was associated with enhanced activation and cytokine production by CD8^+^ T cells and enhanced deposition of tumor-specific Ig in the tumor bed. Furthermore, a phase I/II clinical trial demonstrated that lymphoma patients that received combined radiation and TLR9 agonist had improved clinical responses suggesting that supplemental danger signals in the context of tumor irradiation my drive more potent host T cell responses (Brody et al., 2010). Experiments that further elucidate both the unique and overlapping aspects of radiation-induced danger signals and exogenous adjuvants on host T cell activation and priming are needed.

## T CELL PRIMING FOLLOWING TREATMENT OF ESTABLISHED TUMORS WITH LOCAL RT

Data in preclinical models have demonstrated increased priming of tumor antigen-specific CD8^+^ T cells in the draining lymph node (dLN) several days following treatment of established tumors with ablative single dose local RT (Lugade et al., 2005; Lee et al., 2009). Particularly, our group demonstrated enhanced cross-presentation of tumor antigen by CD11c^+^ DCs present in the dLN following migration from the tumor (Lee et al., 2009). Recently, this mechanism was expanded to incorporate proximal events in the tumor microenvironment. Local radiation has been shown to induced rapid recruitment and infiltration of leukocytes (Shiao and Coussens, 2010; Burnette et al., 2011). Among the recruited cells, circulating monocytes can give rise to CD11c^+^ DCs. Within the irradiated tumor microenvironment these DCs encounter myriad danger signals and capture antigens from dying tumor cells through phagocytic receptors (discussed above). Our group demonstrated that treatment of tumors with local ablative RT could greatly enhance the cross-priming capacity of tumor-infiltrating DCs (TIDCs), and this effect was shown to be critically dependent on type I interferon (IFN) signaling in bone marrow-derived hematopoietic cells (Burnette et al., 2011). Importantly, the enhanced cross-priming capacity of TIDC was not simply dependent on availability of newly liberated tumor antigen, but rather was dependent on signals unique to the irradiated tumor microenvironment. The development of DCs in the irradiated tumor microenvironment that are competent to prime tumor antigen-specific T cells precedes the enhanced T cell priming that we and others have observed in the dLN. These sequential observations suggest that migration of functional TIDCs to the dLN drives T cell priming following local RT (Lugade et al., 2008; Meng et al., 2010; Gupta et al., 2012). Interestingly, type I IFN was shown to be a critical mediator of spontaneous tumor antigen-specific CD8^+^ T cell priming (Fuertes et al., 2011). More specifically, DCs were shown to be the essential targets of type I IFN signaling to mediated T cell priming and drive tumor immunoediting (Diamond et al., 2011). Together these results paint a compelling picture that local radiation may, in fact, rekindle central aspects of innate and adaptive immunity to induce subsequent rounds of immunoediting, and in some cases, complete regression.

## T CELL MIGRATION AND EFFECTOR FUNCTION IN THE TUMOR MICROENVIRONMENT

The tumor microenvironment represents a formidable challenge to immune-mediated recognition and killing of tumor cells. In the absence of local RT, strategies aimed at increasing the pool of tumor antigen-specific T cells, such as therapeutic vaccination or adoptive transfer of large numbers of specific T cells, fails to exert significant effects on tumor outgrowth. However, combining these strategies with local RT can yield impressive results in preclinical models (Harris et al., 2008; Takeshima et al., 2010). Therefore, the capacity of local RT to support immune-mediated tumor regression extends far beyond the effects of local RT on T cell priming, and involves local changes that reinforce immunity subsequent to priming. Local radiation has been shown to facilitate the recruitment of activated T cells to the tumor and induce changes in the local microenvironment and on tumor cell themselves that can greatly enhance T cell effector function. RT can upregulate expression of adhesion molecules, such as VCAM-1, E-selectin, and ICAM-1, by vascular endothelial cells within the tumor and induce expression of T cell chemokines that promote T cell adhesion and extravasation into the tumor microenvironment (Handschel et al., 1999; Lugade et al., 2008). Both the expression of adhesion molecules and the production of T cell attractive chemokines are likely a product of a feedforward mechanism induced by production of IFNs in the tumor microenvironment (Lugade et al., 2008; Meng et al., 2010). In addition, tumor cells can directly produce CXCL16 following irradiation leading to the recruitment of activated CD8^+^ CXCR6^+^ effector cells (Matsumura et al., 2008; Matsumura and Demaria, 2010). Furthermore, RT has been shown to induce several changes that directly affect the ability of effector T cells to efficiently recognize and kill tumor cells.

As previously noted, radiation increases surface expression of MHC on tumor cells, which increases the likelihood of a productive interaction with cognate antigen-specific T cells. Upregulation of MHC likely occurs through several mechanisms that coordinately drive robust expression. In addition to the mechanism proposed by Reits et al. (2006; discussed above), local radiation has been shown to induce MHC expression through induction of IFN-β that can signal to tumor cells in an autocrine/paracrine fashion (Wan et al., 2012). IFN-γ secretion by infiltrating effector cells can also further augment MHC expression and promote T cell-mediated recognition of tumor cells through cognate TCR:peptide/MHC interactions. Bolstering the direct TCR-mediated recognition of tumor cells, is the local expression of ligands for the NKG2D activating receptor. NKG2D ligands have been shown to be expressed as a consequence of cellular transformation, are upregulated by cellular stress, and directly induced by irradiation through activation of the DNA damage pathway (Gasser et al., 2005). NKG2D is an activating receptor expressed by NK cells and activated CD8^+^ T cells that, upon engagement, can significantly increase cytolytic potential (Markiewicz et al., 2005; González et al., 2008; Champsaur and Lanier, 2010). Upregulation of NKG2D ligands could be a robust mechanism for local restimulation of CTL and enhanced cytokine production, however, the mechanisms regulating expression are complex and expression among tumors is variable (Nausch and Cerwenka, 2008). Finally, radiation can upregulate expression of the FAS death receptor on tumor cells to induce sensitivity to T cell expressed FAS ligand (Chakraborty et al., 2004). The induced sensitivity of tumor cells to FAS-mediated killing represents a TCR-independent mechanism for tumor cell killing and can function as a more potent cytotoxic modality especially in instances where TCR affinity is low and perforin-mediated cytotoxicity is less efficient (Kessler et al., 1998). The combination of these local effects likely accounts for a significant portion of the interaction with immunotherapy.

## SYNERGY WITH IMMUNOTHERAPY

The immune modulating capacity of RT is clearly multifaceted and can, in some preclinical models, lead to robust anti-tumor immunity that can mediate complete tumor regression as a single modality. However, it has been reported that radiation could increase some immunosuppressive aspects of the tumor microenvironment such as regulatory T cell (Treg) accumulation depending on the dose and timing (Kachikwu et al., 2011; Schaue et al., 2011). Therefore, in order to maximize the immunostimulatory effects of RT, strategies that combine local RT with immunotherapy are required to generate durable T cells responses in patients. Among the prospects for targeted therapies that can directly enhance T cell responses, monoclonal antibodies that modulate T cell coactivating and coinhibitory receptors, or their ligands, are the most accessible. Productive T cell priming and the induction of tolerance are determined by a complex integration of many stimulatory and inhibitory receptors that reinforce and dampen the primary TCR:peptide/MHC interaction, respectively. Recently, a monoclonal antibody targeting the T cell negative regulator, CTLA-4, received FDA approval following a proven survival benefit in a randomized clinical trial of patients with metastatic melanoma (Hodi et al., 2010). CTLA-4 is expressed by activated T cells and functions as a natural regulatory mechanism to dampen T cell activation and prevent autoimmunity by competitively inhibiting the interaction of CD28 on T cells with B7-1/B7-2 on APCs (Rudd et al., 2009). CD28 cosignaling is required for optimal induction of CD25, which together with IL-2Rβ and common gamma chain, form the high-affinity IL-2 receptor. IL-2 signaling induces both the differentiation and survival of effector T cells, and the inability to upregulate CD25, and therefore respond to IL-2, is associated with T cell anergy and tolerance. In addition, Tregs constitutively express CTLA-4, which has been shown to directly control DC maturation and the induction of T cell tolerance by downregulating B7-1/B7-2 expression on DCs to block the CD28:B7-1/B7-2 signal (Wing et al., 2008; Qureshi et al., 2011).

An accepted hypothesis is that anti-CTLA-4 blocking antibodies promote enhanced T cell activation and proliferation to promote effector cell priming (Chambers et al., 2001; Pardoll and Drake, 2012). In preclinical models, local RT and CTLA-4 blockade was shown to mediate synergistic effects (Dewan et al., 2009). Furthermore, in mice concurrently challenged with two tumors, treatment of one tumor with local RT in combination with systemic administration of anti-CTLA-4 could induce significant growth delay in the second tumor that did not receive local RT; a process referred to as the abscopal effect (Dewan et al., 2009). The precise mechanism underlying the abscopal regression of unirradiated tumors was not investigated, but the results are consistent with increased priming of tumor antigen-specific T cells that subsequently infiltrate the tumor. Such an effect would likely be mediated by blocking the engagement of CTLA-4 on effector T cells in the context of heightened cross-priming capacity of DCs in the dLN (discussed above). Interestingly, data from Dewan et al. (2009) also reported that a fractionated dose of 8 Gy × 3 was optimal for induction of an abscopal effect when combined with anti-CTLA-4, whereas and abscopal effect was not observed when tumors were treated with 20 Gy × 1 or 6 Gy × 5 alone or in combination with anti-CTLA-4. Although the authors refer to 8 Gy × 3 as a fractionated schedule, this treatment scheme is probably more accurately described as hypofractionation. The precise mechanistic basis for the ability of 8 Gy × 3 to properly synergize with anti-CTLA-4 was not explored, however, the authors did note that this dose scheme did result in the highest level of infiltration and IFN-γ production by T cells. The synergy between local RT and CTLA-4 blockade observed in preclinical models appears to translate well into the clinic. Several reports in melanoma patients have demonstrated abscopal regression following treatment with local RT and anti-CTLA-4 (ipilimumab) that was associated with elevated immunity to tumor-associated antigens (Postow et al., 2012; Stamell et al., 2012). At present, there is no clear role for CTLA-4 blockade in the tumor microenvironment. It is reasonable to suspect that Tregs expressing CTLA-4 in the tumor microenvironment could similarly modulate DCs that infiltrate the tumor, however, definitive evidence that CTLA-4 participates in Treg-mediated suppression in the tumor microenvironment is lacking. Strategies that enhance T cell activation by engaging costimulatory receptors expressed on T cells represent a complimentary approach to blockade of negative regulators.

Enhancement of effector cell priming is a shared mechanism between anti-CTLA-4 and other targeted therapies employing agonistic antibodies against the costimulatory receptors OX40, 4-1BB (CD137), and CD27 (for a detailed review, see Redmond et al., 2009). Briefly, stimulation of T cells through OX40 results in enhanced T cell activation and effector cell differentiation, in part, through enhancing the expression of CD25 and promoting T cell sensitivity to IL-2. A recent report demonstrated that agonistic OX40 antibodies in combination with systemic IL-2 administration could generate potent anti-tumor immunity, and the synergistic nature of the combination resulted from the ability of systemic IL-2 to upregulate OX40 expression on activated T cells (Redmond et al., 2012).

Results from a phase I study of stereotactic body radiation therapy (SBRT) and systemic IL-2 in melanoma and renal cell carcinoma demonstrated that this combination could result in impressive responses in both tumor types (Seung et al., 2012). Addition of OX40 agonistic antibody to this clinical protocol would be predicted to further enhance responses and perhaps increase the rate of complete response. Based on these results, it seems likely that a natural synergy might exist between agonistic OX40 antibodies and anti-CTLA-4 to induce optimal expression of CD25 and OX40 and maximize effector T cell differentiation. Agonistic OX40 antibody has also been shown to synergize with high dose local RT (20 Gy × 3) and was associated with enhanced expression of CD25 by tumor-infiltrating CD8^+^ T cells (Gough et al., 2010). Importantly, OX40 stimulation possesses no inherent ability to polarize T cells toward one particular effector subset, but rather, drives T cell polarization in the context of the inflammatory milieu. Considering the nature of most tumor-associated antigens, it is important to note that costimulation through OX40 can rescue priming of low avidity T cells, and can also reverse T cell tolerance against self-antigens. Taken together, the mixed preclinical and clinical data employing local ablative RT with OX40 agonistic antibody, systemic IL-2, or anti-CTLA-4 antibody demonstrate that signaling through CD25 and OX40 reciprocally reinforce each other to augment effector cell priming initiated by local RT and improve the quality and magnitude of T cell responses against tumor-associated antigens. Future clinical trials that employ local RT, anti-CTLA-4, and agonistic OX86 are likely to yield impressive results.

In addition to the goal of improving T cell activation and effector cell generation, strategies that target immune suppressive mechanism in the tumor microenvironment are equally important. Local RT does have the ability to modify the tumor microenvironment, however, many tumors exploit natural immune regulatory mechanisms to subvert induced T cells responses. Expression of programmed death ligand-1 (PD-L1) in the tumor microenvironment can deliver an inhibitory signal through it’s receptor PD-1 that is expressed on a majority of activated effector T cells. PD-L1 expression has been observed across many tumor types (Zou and Chen, 2008) where it mediates apoptosis of infiltrating T cells leading to tumor immune evasion (Dong et al., 2002). Interestingly, PD-L1 expression can be directly induced by IFNs indicating that effector T cell activity within the tumor microenvironment can initiate PD-L1 expression as a negative feedback loop to squelch T cell effector function (Lee et al., 2006). Corroborating the role of effector T cell-mediated PD-L1 upregulation, a recent study in human melanoma demonstrated a strong correlation between PD-L1 expression and intratumoral T cell infiltration and IFN-γ (Taube et al., 2012). Data in preclinical models suggest that PD-L1 blockade is necessary in some circumstances to fully uncover anti-tumor immunity that is induced by local RT in combination with costimulatory receptor engagement. Verbrugge et al. (2012) demonstrated that local RT combined with anti-OX40 could mediate significant growth delay of orthotopic AT-3 mammary tumors, however, the addition of anti-PD-L1 was required to mediate complete tumor regression. Future studies will likely continue to uncover optimal combinatorial strategies that enhance the effects of local RT during each phase of the T cell response.

## ADDITIONAL CONSIDERATIONS AND CONCLUDING REMARKS

From the data discussed above it is clear that combination strategies employing high dose ablative RT and immunotherapy hold a lot of promise for improving anti-tumor immunity and mediating complete tumor regression. There are many important outstanding questions before RT and immunotherapy can reliably be combined in cancer therapy. Amongst the most important are what is (are) the optimal fractionation (dose delivery of radiation) schemes to increase anti-tumor immunity? Does daily fractionation continuously kill infiltrating T cells and/or reduce their function, or are tumor-infiltrating activated T cells functionally resistant to the low doses employed in traditional fractionation or hyperfractionated schedules? Does inclusion of the dLNs suppress or enhance the immunogenic effects of radiation, and does the timing of dLN irradiation change the response to treatment or therapeutic vaccination. What is the optimal “immune activating” strategy, e.g., high dose cytokines, vaccination, etc.? Answers to these and other questions may improve the local effects of RT and help to understand the basis of activating and improving the local tumor response to RT and the abscopal effect against distant metastasis.

## Conflict of Interest Statement

The authors declare that the research was conducted in the absence of any commercial or financial relationships that could be construed as a potential conflict of interest.

## References

[B1] Ahrens S. Zelenay S. Sancho D. Hanč P. Kjær S. Feest C. (2012). F-actin is an evolutionarily conserved damage-associated molecular pattern recognized by DNGR-1, a receptor for dead cells. *Immunity* 36 635–645 22483800 10.1016/j.immuni.2012.03.008

[B2] Apetoh L. Ghiringhelli F. Tesniere A. Obeid M. Ortiz C. Criollo A. (2007). Toll-like receptor 4-dependent contribution of the immune system to anticancer chemotherapy and radiotherapy. *Nat. Med.* 13 1050–1059 17704786 10.1038/nm1622

[B3] Bianchi M. E. (2006). DAMPs, PAMPs and alarmins: all we need to know about danger. *J. Leukoc. Biol.* 81 1–5 17032697 10.1189/jlb.0306164

[B4] Bianchi M. E. Manfredi A. A. (2007). High-mobility group box 1 (HMGB1) protein at the crossroads between innate and adaptive immunity. *Immunol. Rev.* 220 35–46 17979838 10.1111/j.1600-065X.2007.00574.x

[B5] Boon T. (1996). Human tumor antigens recognized by T lymphocytes. *J. Exp. Med.* 183 725–729 8642276 10.1084/jem.183.3.725 PMC2192342

[B6] Brody J. D. Ai W. Z. Czerwinski D. K. Torchia J. A. Levy M. Advani R. H. (2010). In situ vaccination with a TLR9 agonist induces systemic lymphoma regression: a phase I/II study. *J. Clin. Oncol.* 28 4324–4332 20697067 10.1200/JCO.2010.28.9793 PMC2954133

[B7] Burnette B. C. Liang H. Lee Y. Chlewicki L. Khodarev N. N. Weichselbaum R. R. (2011). The efficacy of radiotherapy relies upon induction of type I interferon-dependent innate and adaptive immunity. *Cancer Res.* 71 2488–2496 21300764 10.1158/0008-5472.CAN-10-2820 PMC3070872

[B8] Chakraborty M. Abrams S. I. Coleman C. N. Camphausen K. Schlom J. Hodge J. W. (2004). External beam radiation of tumors alters phenotype of tumor cells to render them susceptible to vaccine-mediated T-cell killing. *Cancer Res.* 64 4328–4337 15205348 10.1158/0008-5472.CAN-04-0073

[B9] Chambers C. A. Kuhns M. S. Egen J. G. Allison J. P. (2001). CTLA-4-mediated inhibition in regulation of T cell responses: mechanisms and manipulation in tumor immunotherapy. *Annu. Rev. Immunol.* 19 565–594 11244047 10.1146/annurev.immunol.19.1.565

[B10] Champsaur M. Lanier L. L. (2010). Effect of NKG2D ligand expression on host immune responses. *Immunol. Rev.* 235 267–285 20536569 10.1111/j.0105-2896.2010.00893.x PMC2885032

[B11] Dewan M. Z. Galloway A. E. Kawashima N. DeWyngaert J. K. Babb J. S. Formenti S. C. (2009). Fractionated but not single-dose radiotherapy induces an immune-mediated abscopal effect when combined with anti-CTLA-4 antibody. *Clin. Cancer Res.* 15 5379–5388 19706802 10.1158/1078-0432.CCR-09-0265 PMC2746048

[B12] Diamond M. S. Kinder M. Matsushita H. Mashayekhi M. Dunn G. P. Archambault J. M. (2011). Type I interferon is selectively required by dendritic cells for immune rejection of tumors. *J. Exp. Med.* 208 1989–2003 21930769 10.1084/jem.20101158 PMC3182061

[B13] Dong H. Strome S. E. Salomao D. R. Tamura H. Hirano F. Flies D. B. (2002). Tumor-associated B7-H1 promotes T-cell apoptosis: a potential mechanism of immune evasion. *Nat. Med.* 8 793–800 12091876 10.1038/nm730

[B14] Dunn G. P. Koebel C. M. Schreiber R. D. (2006). Interferons, immunity and cancer immunoediting. *Nat. Rev. Immunol.* 6 836–848 17063185 10.1038/nri1961

[B15] Elliott M. R. Chekeni F. B. Trampont P. C. Lazarowski E. R. Kadl A. Walk S. F. (2009). Nucleotides released by apoptotic cells act as a find-me signal to promote phagocytic clearance. *Nature* 461 282–286 19741708 10.1038/nature08296 PMC2851546

[B16] Engelhard V. H. Bullock T. N. J. Colella T. A. Sheasley S. L. Mullins D. W. (2002). Antigens derived from melanocyte differentiation proteins: self-tolerance, autoimmunity, and use for cancer immunotherapy. *Immunol. Rev.* 188 136–146 12445287 10.1034/j.1600-065x.2002.18812.x

[B17] Eriksson D. Stigbrand T. (2010). Radiation-induced cell death mechanisms. *Tumor Biol.* 31 363–372 10.1007/s13277-010-0042-8 20490962

[B18] Fuertes M. B. Kacha A. K. Kline J. Woo S.-R. Kranz D. M. Murphy K. M. (2011). Host type I IFN signals are required for antitumor CD8^+^ T cell responses through CD8α^+^ dendritic cells. *J. Exp. Med.* 208 2005–2016 21930765 10.1084/jem.20101159 PMC3182064

[B19] Gasser S. Orsulic S. Brown E. J. Raulet D. H. (2005). The DNA damage pathway regulates innate immune system ligands of the NKG2D receptor. *Nature* 436 1186–1190 15995699 10.1038/nature03884 PMC1352168

[B20] Ghiringhelli F. Apetoh L. Tesniere A. Aymeric L. Ma Y. Ortiz C. (2009). Activation of the NLRP3 inflammasome in dendritic cells induces IL-1beta-dependent adaptive immunity against tumors. *Nat. Med.* 15 1170–1178 19767732 10.1038/nm.2028

[B21] González S. López-Soto A. Suarez-Alvarez B. López-Vázquez A López-Larrea C. (2008). NKG2D ligands: key targets of the immune response. *Trends Immunol.* 29 397–403 18602338 10.1016/j.it.2008.04.007

[B22] Gough M. J. Crittenden M. R. Sarff M. Pang P. Seung S. K. Vetto J. T. (2010). Adjuvant therapy with agonistic antibodies to CD134 (OX40) increases local control after surgical or radiation therapy of cancer in mice. *J. Immunother.* 33 798–809 20842057 10.1097/CJI.0b013e3181ee7095 PMC3563298

[B23] Gupta A. Probst H. C. Vuong V. Landshammer A. Muth S. Yagita H. (2012). Radiotherapy promotes tumor-specific effector CD8+ T cells via dendritic cell activation. *J. Immunol.* 189 558–566 22685313 10.4049/jimmunol.1200563

[B24] Handschel J. Prott F. J. Sunderkötter C. Metze D. Meyer U. Joos U. (1999). Irradiation induces increase of adhesion molecules and accumulation of beta2-integrin-expressing cells in humans. *Int. J. Radiat. Oncol. Biol. Phys.* 45 475–481 10487574 10.1016/s0360-3016(99)00202-3

[B25] Harris T. J. Hipkiss E. L. Borzillary S. Wada S. Grosso J. F. Yen H.-R. (2008). Radiotherapy augments the immune response to prostate cancer in a time-dependent manner. *Prostate* 68 1319–1329 18561247 10.1002/pros.20794 PMC2710770

[B26] Hodi F. S. O’Day S. J. McDermott D. F. Weber R. W. Sosman J. A. Haanen J. B. (2010). Improved survival with ipilimumab in patients with metastatic melanoma. *N. Engl. J. Med.* 363 711–723 20525992 10.1056/NEJMoa1003466 PMC3549297

[B27] Jäger D. Jäger E. Knuth A. (2001). Immune responses to tumour antigens: implications for antigen specific immunotherapy of cancer. *J. Clin. Pathol.* 54 669–674 11533070 10.1136/jcp.54.9.669 PMC1731514

[B28] Kachikwu E. L. Iwamoto K. S. Liao Y.-P. DeMarco J. J. Agazaryan N. Economou J. S. (2011). Radiation enhances regulatory T cell representation. *Int. J. Radiat. Oncol. Biol. Phys.* 81 1128–1135 21093169 10.1016/j.ijrobp.2010.09.034 PMC3117954

[B29] Kessler B. Hudrisier D. Schroeter M. Tschopp J. Cerottini J. C. Luescher I. F. (1998). Peptide modification or blocking of CD8, resulting in weak TCR signaling, can activate CTL for Fas- but not perforin-dependent cytotoxicity or cytokine production. *J. Immunol.* 161 6939–6946 9862728

[B30] Lee S.-J. Jang B.-C. Lee S.-W. Yang Y.-I. Suh S.-I. Park Y.-M. (2006). Interferon regulatory factor-1 is prerequisite to the constitutive expression and IFN-gamma-induced upregulation of B7-H1 (CD274). *FEBS Lett.* 580 755–762 16413538 10.1016/j.febslet.2005.12.093

[B31] Lee Y. Auh S. L. Wang Y. Burnette B. Wang Y. Meng Y. (2009). Therapeutic effects of ablative radiation on local tumor require CD8+ T cells: changing strategies for cancer treatment. *Blood* 114 589–595 19349616 10.1182/blood-2009-02-206870 PMC2713472

[B32] Lugade A. A. Moran J. P. Gerber S. A. Rose R. C. Frelinger J. G. Lord E. M. (2005). Local radiation therapy of B16 melanoma tumors increases the generation of tumor antigen-specific effector cells that traffic to the tumor. *J. Immunol.* 174 7516–7523 15944250 10.4049/jimmunol.174.12.7516

[B33] Lugade A. A. Sorensen E. W. Gerber S. A. Moran J. P. Frelinger J. G. Lord E. M. (2008). Radiation-induced IFN-gamma production within the tumor microenvironment influences antitumor immunity. *J. Immunol.* 180 3132–3139 18292536 10.4049/jimmunol.180.5.3132

[B34] Markiewicz M. A. Carayannopoulos L. N. Naidenko O. V. Matsui K. Burack W. R. Wise E. L. (2005). Costimulation through NKG2D enhances murine CD8+ CTL function: similarities and differences between NKG2D and CD28 costimulation. *J. Immunol.* 175 2825–2833 16116168 10.4049/jimmunol.175.5.2825

[B35] Matsumura S. Demaria S. (2010). Up-regulation of the pro-inflammatory chemokine CXCL16 is a common response of tumor cells to ionizing radiation. *Radiat. Res.* 173 418–425 20334513 10.1667/RR1860.1 PMC2857712

[B36] Matsumura S. Wang B. Kawashima N. Braunstein S. Badura M. Cameron T. O. (2008). Radiation-induced CXCL16 release by breast cancer cells attracts effector T cells. *J. Immunol.* 181 3099–3107 18713980 10.4049/jimmunol.181.5.3099 PMC2587101

[B37] Matzinger P. (2002). The danger model: a renewed sense of self. *Science* 296 301–305 11951032 10.1126/science.1071059

[B38] Matzinger P. (1994). Tolerance, danger, and the extended family. *Annu. Rev. Immunol.* 12 991–1045 8011301 10.1146/annurev.iy.12.040194.005015

[B39] Meng Y. Mauceri H. J. Khodarev N. N. Darga T. E. Pitroda S. P. Beckett M. A. (2010). Ad.Egr-TNF and local ionizing radiation suppress metastases by interferon-beta-dependent activation of antigen-specific CD8+ T cells. *Mol. Ther.* 18 912–920 20197756 10.1038/mt.2010.18 PMC2890103

[B40] Nausch N. Cerwenka A. (2008). NKG2D ligands in tumor immunity. *Oncogene* 27 5944–5958 18836475 10.1038/onc.2008.272

[B41] Obeid M. Panaretakis T. Joza N. Tufi R. Tesniere A. van Endert P. (2007). Calreticulin exposure is required for the immunogenicity of gamma-irradiation and UVC light-induced apoptosis. *Cell Death Differ.* 14 1848–1850 17657249 10.1038/sj.cdd.4402201

[B42] Obeid M. Tesniere A. Ghiringhelli F. Fimia G. M. Apetoh L. Perfettini J.-L. (2006). Calreticulin exposure dictates the immunogenicity of cancer cell death. *Nat. Med.* 13 54–61 17187072 10.1038/nm1523

[B43] Pardoll D. Drake C. (2012). Immunotherapy earns its spot in the ranks of cancer therapy. *J. Exp. Med.* 209 201–209 22330682 10.1084/jem.20112275 PMC3280881

[B44] Postow M. A. Callahan M. K. Barker C. A. Yamada Y. Yuan J. Kitano S. (2012). Immunologic correlates of the abscopal effect in a patient with melanoma. *N. Engl. J. Med.* 366 925–931 22397654 10.1056/NEJMoa1112824 PMC3345206

[B45] Poulin L. F. Reyal Y. Uronen-Hansson H. Schraml B. Sancho D. Murphy K. M. (2012). DNGR-1 is a specific and universal marker of mouse and human Batf3-dependent dendritic cells in lymphoid and non-lymphoid tissues. *Blood* 119 6052–6062 22442345 10.1182/blood-2012-01-406967

[B46] Poulin L. F. Salio M. Griessinger E. Anjos-Afonso F. Craciun L. Chen J. L. (2010). Characterization of human DNGR-1+ BDCA3+ leukocytes as putative equivalents of mouse CD8+ dendritic cells. *J. Exp. Med.* 207 1261–1271 20479117 10.1084/jem.20092618 PMC2882845

[B47] Qureshi O. S. Zheng Y. Nakamura K. Attridge K. Manzotti C. Schmidt E. M. (2011). Trans-endocytosis of CD80 and CD86: a molecular basis for the cell-extrinsic function of CTLA-4. *Science* 332 600–603 21474713 10.1126/science.1202947 PMC3198051

[B48] Redmond W. L. Ruby C. E. Weinberg A. D. (2009). The role of OX40-mediated co-stimulation in T-cell activation and survival. *Crit. Rev. Immunol.* 29 187–201 19538134 10.1615/critrevimmunol.v29.i3.10 PMC3180959

[B49] Redmond W. L. Triplett T. Floyd K. Weinberg A. D. (2012). Dual anti-OX40/IL-2 therapy augments tumor immunotherapy via IL-2R-mediated regulation of OX40 expression. *PLoS ONE* 7 e34467 10.1371/journal.pone.0034467 PMC3319580 22496812

[B50] Reits E. A. Hodge J. W. Herberts C. A. Groothuis T. A. Chakraborty M. Wansley E. K. (2006). Radiation modulates the peptide repertoire, enhances MHC class I expression, and induces successful antitumor immunotherapy. *J. Exp. Med.* 203 1259–1271 16636135 10.1084/jem.20052494 PMC3212727

[B51] Rudd C. E. Taylor A. Schneider H. (2009). CD28 and CTLA-4 coreceptor expression and signal transduction. *Immunol. Rev.* 229 12–26 19426212 10.1111/j.1600-065X.2009.00770.x PMC4186963

[B52] Rupnow B. A. Knox S. J. (1999). The role of radiation-induced apoptosis as a determinant of tumor responses to radiation therapy. *Apoptosis* 4 115–143 14634289 10.1023/a:1009675028784

[B53] Sancho D. Joffre O. P. Keller A. M. Rogers N. C. Martínez D. Hernanz-Falcón P. (2009). Identification of a dendritic cell receptor that couples sensing of necrosis to immunity. *Nature* 458 899–903 19219027 10.1038/nature07750 PMC2671489

[B54] Santin A. D. Hermonat P. L. Ravaggi A. Chiriva-Internati M. Pecorelli S. Parham G. P. (1998). Radiation-enhanced expression of E6/E7 transforming oncogenes of human papillomavirus-16 in human cervical carcinoma. *Cancer* 83 2346–2352 9840534 10.1002/(sici)1097-0142(19981201)83:11<2346::aid-cncr14>3.0.co;2-g

[B55] Scanlan M. J. Gure A. O. Jungbluth A. A. Old L. J. Chen Y.-T. (2002). Cancer/testis antigens: an expanding family of targets for cancer immunotherapy. *Immunol. Rev.* 188 22–32 12445278 10.1034/j.1600-065x.2002.18803.x

[B56] Schaue D. Ratikan J. A. Iwamoto K. S. McBride W. H. (2011). Maximizing tumor immunity with fractionated radiation. *Int. J. Radiat. Oncol. Biol. Phys.* 83 1306–1310 22208977 10.1016/j.ijrobp.2011.09.049 PMC3337972

[B57] Schreibelt G. Klinkenberg L. J. J. Cruz L. J. Tacken P. J. Tel J. Kreutz M. (2012). The C-type lectin receptor CLEC9A mediates antigen uptake and (cross-) presentation by human blood BDCA3+ myeloid dendritic cells. *Blood* 119 2284–2292 22234694 10.1182/blood-2011-08-373944

[B58] Seung S. K. Curti B. D. Crittenden M. Walker E. Coffey T. Siebert J. C. (2012). Phase 1 study of stereotactic body radiotherapy and interleukin-2 – tumor and immunological responses. *Sci. Transl. Med. * 4 137ra74 10.1126/scitranslmed.3003649 22674552

[B59] Sharma A. Bode B. Wenger R. H. Lehmann K. Sartori A. A. Moch H. (2011). γ-Radiation promotes immunological recognition of cancer cells through increased expression of cancer-testis antigens in vitro and in vivo. *PLoS ONE* 6 e28217 10.1371/journal.pone.0028217 PMC3226680 22140550

[B60] Shiao S. L. Coussens L. M. (2010). The tumor-immune microenvironment and response to radiation therapy. *J. Mammary Gland Biol. Neoplasia* 15 411–421 21161342 10.1007/s10911-010-9194-9 PMC3011087

[B61] Stamell E. F. Wolchok J. D. Gnjatic S. Lee N. Y. Brownell I. (2012). The abscopal effect associated with a systemic anti-melanoma immune response. *Int. J. Radiat. Oncol. Biol. Phys.* (in press) 10.1016/j.ijrobp.2012.03.017 PMC3415596 22560555

[B62] Takeshima T. Chamoto K. Wakita D. Ohkuri T. Togashi Y. Shirato H. (2010). Local radiation therapy inhibits tumor growth through the generation of tumor-specific CTL: its potentiation by combination with Th1 cell therapy. *Cancer Res.* 70 2697–2706 20215523 10.1158/0008-5472.CAN-09-2982

[B63] Taube J. M. Anders R. A. Young G. D. Xu H. Sharma R. McMiller T. L. (2012). Colocalization of inflammatory response with B7-h1 expression in human melanocytic lesions supports an adaptive resistance mechanism of immune escape. *Sci. Transl. Med.* 4 127ra37 10.1126/scitranslmed.3003689 PMC3568523 22461641

[B64] Uchi H. Stan R. Turk M. J. Engelhorn M. E. Rizzuto G. A. Goldberg S. M. (2006). Unraveling the complex relationship between cancer immunity and autoimmunity: lessons from melanoma and vitiligo. *Adv. Immunol.* 90 215–241 16730265 10.1016/S0065-2776(06)90006-6

[B65] Verbrugge I. Hagekyriakou J. Sharp L. L. Galli M. West A. C. McLaughlin N. M. (2012). Radiotherapy increases the permissiveness of established mammary tumors to rejection by immunomodulatory antibodies. *Cancer Res.* 72 3163–3174 22570253 10.1158/0008-5472.CAN-12-0210

[B66] Wan S. Pestka S. Jubin R. G. Lyu Y. L. Tsai Y.-C. Liu L. F. (2012). Chemotherapeutics and radiation stimulate MHC class I expression through elevated interferon-beta signaling in breast cancer cells. *PLoS ONE* 7 e32542 10.1371/journal.pone.0032542 PMC3291570 22396773

[B67] Wing K. Onishi Y. Prieto-Martin P. Yamaguchi T. Miyara M. Fehervari Z. (2008). CTLA-4 control over Foxp3+ regulatory T cell function. *Science* 322 271–275 18845758 10.1126/science.1160062

[B68] Zhang H. Liu L. Yu D. Kandimalla E. R. Sun H. B. Agrawal S. (2012). An in situ autologous tumor vaccination with combined radiation therapy and TLR9 agonist therapy. *PLoS ONE* 7 e38111 10.1371/journal.pone.0038111 PMC3364192 22666458

[B69] Zou W. Chen L. (2008). Inhibitory B7-family molecules in the tumour microenvironment. *Nat. Rev. Immunol.* 8 467–477 18500231 10.1038/nri2326

